# Converting acute inpatient take to outpatient take with fast-track assessment in internal medicine wards – a before-after study

**DOI:** 10.1186/s12913-019-4175-1

**Published:** 2019-05-31

**Authors:** Cathrine Bell, Ulrich Fredberg, Anders Damgaard Moeller Schlünsen, Peter Vedsted

**Affiliations:** 1 Diagnostic Centre, University Research Clinic for Innovative Patient Pathways, Silkeborg Regional Hospital, Aarhus University, Falkevej 1-3, 8600 Silkeborg, Denmark; 2 Research Unit for General Practice, Faculty of Health, Aarhus University, Aarhus, Denmark

**Keywords:** Outpatient, Acute care, Bed occupancy, Admission, Patient pathway

## Abstract

**Background:**

With an extensive rise in the number of acute patients and increases in both admissions and readmissions, hospitals are at times overcrowded and under immense pressure and this may challenge patient safety. This study evaluated an innovative strategy converting acute internal medicine inpatient take to an outpatient take. Here, acute patients, following referral, underwent fast-track assessment to the needed level of medical care as outpatients, directly in internal medicine wards.

**Method:**

The two internal medicine wards at Diagnostic Centre, Silkeborg, Denmark, changed their take of acute patients 1st of March 2017. The intervention consisted of acute medical patients being received in medical examination chairs, going through accelerated evaluation as outpatients with assessment within one hour for either admission or another form of treatment. A before-and-after study design was used to evaluate changes in activity. All referred patients for 10 months following implementation of the intervention were compared with patients referred in corresponding months the previous year.

**Results:**

A total of 5339 contacts (3632 patients) who underwent acute medical assessment (2633 contacts before and 2706 after) were included. Median hospital length-of-stay decreased from 32.6 h to 22.3 h, and the proportion of referred acute patients admitted decreased with 36.3% points from 94.5 to 58.2%. The median length-of-admission time for the admitted patients increased as expected after the intervention. The risk of being admitted, being readmitted as well as having a hospital length-of-time longer than 24 h, 72 h or 7 days, respectively, were significantly lower during the after-period in comparison to the before-period. Adverse effects, unplanned re-contacts, total contacts to general practice and mortality did not change after the intervention.

**Conclusion:**

Assessing referred acute patients in medical examination chairs as outpatients directly in internal medicine wards and promoting an accelerated trajectory, reduced inpatient admissions and total length-of-stay considerably. This strategy seems effective in everyday acute medical patients and has the potential to ease the increasing pressure on the acute take for wards receiving acute medical patients.

## Background

Overcrowding in and readmission to hospitals is of global concern. Acute medical patients comprise an increasing proportion of admitted patients, which puts medical wards under immense pressure. Acute elderly patients with chronic diseases are at greater risk of admission [1] and constitute a third of acute patients in hospitals [2–4] which is intensified with an aging multimorbid population. Hospital overcrowding is associated with increased admission time and increased inpatient mortality [5]. This emphasises the demand for innovative solutions that can allocate relevant and acute services to patients with acute medical problems.

The traditional model of acute medical care often involves hospital admission. To address the high level of bed occupancy, different entries for acute patients have emerged facilitating more flexible and dynamic bed management [6] with a proliferation of observation and assessment units [7, 8]. These entries comprise a wide variety of forms and allow patients to be observed on a short-term basis without using inpatient facilities [9, 10]. It has been shown that use of acute medical units leads to lower admission rates and length-of-stay without increasing in-hospital mortality [9–12]. However, this type of entry includes an additional step in the trajectory for patients who require consultations with medical specialities and limited evidence exists for reducing hospital admissions along the patient pathway through the emergency department [13].

A different approach has been implemented at the Diagnostic Centre, University Research Clinic for Innovative Patient Pathways in Silkeborg, Denmark. Here, internal medicine wards have converted the entry for referred acute patients to an outpatient take using inpatient facilities. The new trajectory involves choosing the appropriate level of medical care by implementing a fast track evaluation. The evaluation is performed in special medical examination chairs with the aim of completion within one hour assessing whether the patient can be examined and treated as an outpatient or needs admission. We found no studies redirecting referred acute medical patients to accelerated outpatient assessment directly in the medical wards although conversion to an outpatient strategy has been sought-after for different individual diagnosis groups [14, 15]. However, coordinated patient pathways with fewer admissions impact all aspects of health care: by improving both patient satisfaction [9] and conditions for health professionals, by reducing hospital mortality [5, 16], optimising organisational processes and reducing costs [17].

The aim of this study is to analyse differences in acute healthcare utilisations with regard to the number of admissions, length-of-stay, number of contacts with general practice, readmissions and 30-day mortality of referred acute medical patients before and after the acute internal medicine inpatient take was converted to fast track outpatient.

### Setting

Health care in Denmark provides tax-financed universal coverage for all Danish citizens with access to a wide range of health services largely free of charge. Practically, all Danes (99%) are registered with a general practice and general practitioners (GPs) act as coordinators of care to the rest of the health care system [18]. Since 2007, Danish hospitals have been working towards a single entry system for all acute patients via acute departments resembling other countries [8]. However, Silkeborg Regional Hospital has the status of being a development hospital, with the possibility to organise the acute response differently.

At the Silkeborg Regional Hospital, the University Research Clinic for Innovative Patient Pathways within the Diagnostic Centre aims to develop quick and effective patient pathways. The Diagnostic Centre integrates nine medical specialties and radiology. No emergency/observation unit is present. Instead, acute patients are referred (mainly by a GP) directly to medical wards from 8.00 am to 5.00 pm on week days with the exception of persons with signs of ST-elevation myocardial infarction, children, stroke or accidents/emergencies. These patients will be referred to a cardiological department for invasive treatment, neurological department, children’s ward, or to a 24-h emergency department. The Diagnostic Centre covers the entire municipality of Silkeborg located in Central Denmark Region, Denmark, with a catchment area of approx. 70,000 inhabitants aged 18 years or more.

## Methods

### Design and study population

**Fig. 1:**
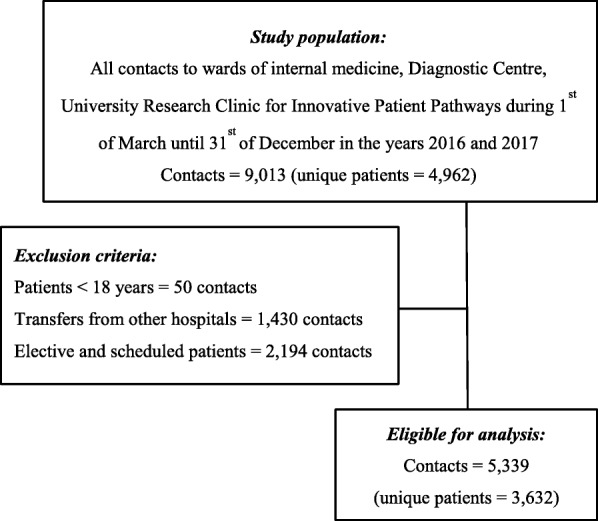
The study population

We conducted a before and after observational study. The study population consisted of all adults from the Municipality of Silkeborg in Denmark referred to the Diagnostic Centre with acute medical indications in comparable months (March –December 2016 and 2017, respectively). Implementation of the new clinical pathway took place 1st of March 2017 and patients referred prior to the intervention constituted the basis of comparison. Patients transferred from other departments, scheduled elective patients, and acute patients from outpatient clinics who came with a treatment plan were excluded (3674 contacts, 40.8%). A remaining 5339 (59.2%) contacts (3632 unique patients), split between 2633 contacts before and 2706 after (Fig. 1) were eligible for analysis.

### Converting the acute medical take

Before, most patients were admitted immediately when received with an acute referral to the internal medicine wards at Silkeborg Regional Hospital. The patient would be assigned a bed on arrival, a junior-physician and nurse-led triage team would evaluate the patient and initiate treatment with the involvement of a specialist-physician, radiologists and laboratory testing when necessary.

The central aspect of the intervention was implementation of the principle considering the patient as an outpatient coming to a fast-track acute assessment of the optimal examination and treatment level. The goal was to ease pressure on the wards and to avoid unnecessary admissions of referred acute patients. Patients remained in their own clothes while evaluation took place in arranged areas with special medical examination chairs suitable for receiving acute patients. These areas were placed in one end of each ward. As before, a junior-physician and nurse-led triage team initiated evaluation of the patient. The intervention included a close collaboration between medical staff, radiology and the laboratory ensuring a quick evaluation. The decision, as to whether to admit the patient (“prescribe a bed”) or send the patient home after investigation and treatment, was made by an internal medical specialist within one hour of the patient’s arrival. Patients could be treated momentarily e.g. with inhalation in the medical examination chair, before a final assessment was made. No specific training was given to any of the intervention providers. A total of 38 stationary beds were reduced to 30 stationary beds and 8 outpatient medical examination chairs. At an overall level, intervention providers were kept regularly informed on their adherence to schedule and use of admissions and fidelity on data registration was upheld by quality support officers.

### Outcomes and covariates

We collected data for the same months before and after the intervention to account for seasonal variation. Data was retrieved for each contact from the hospital patient administration system (PAS), which contains electronic data on each contact to hospitals in the Central Denmark Region. Percentages on inpatient bed occupancy were available on the Business Intelligence Portal for Central Denmark Region. Data on use of general practice in daytime and out-of-hours was collected from the National Health Service Register. Each Danish citizen has a unique personal registration number (PRN), which ensures accurate linkage of information between registers on an individual basis [19, 20].

In both the before and after period, *admissions* were defined by the onset of the actual admission by entries in the hospital’s Electronic Medical Records. *Length-of-admission time* was calculated by the time of admission to the time of discharge according to the Electronic Medical Record. As the new pathway allows for patients spending time at the hospital wards without being admitted, we defined outpatient length-of-stay in both the before and after period as the time from arrival at the hospital to either departure as outpatient or admission. For patients who did not undergo admission, this outpatient time was defined as *hospital length-of-stay*. For patients who were initially received as outpatients and then admitted, *hospital length-of-stay* was measured as the time from arrival as outpatient to discharge as inpatient. *Readmissions* were defined as an admission taking place within 4 h to 30 days after the patient was discharged from the hospital. Hence, readmission only includes patients who first underwent actual inpatient admission. *Re-contacts* was defined as unscheduled hospital contacts taking place within 4 h to 30 days since the last time departing the hospital (either exiting as outpatient or discharged as inpatient). These cut-points were chosen based on the standardized hospital data in Central Region Denmark, where readmission is defined as an admission taking place within 4 h to 30 days from discharge. *Contacts to general practice* were defined as daytime and out-of-hour (4 pm-8 am on weekdays, weekends and bank holidays) contacts within 30 days of leaving the hospital. We accounted for the possibility that a patient might re-enter the hospital within 30 days of last exit and thereby have GP contacts overlapping when counting contacts. From date of death, *mortality* was counted within 30 days of departing the hospital.

*Patient age* (at time of arrival) and gender was retrieved from the patient’s PRN. *Triage,* together with discharge *diagnosis* and registered *comorbidity,* were used to reflect the patient’s health state at arrival. The triage score determining the priority of patients’ treatment was assessed in the wards by nurses and based on the severity of their condition. The score rates from 1 to 5, red corresponding with the highest score of 5. Diagnoses were classified using International Classification of Diseases, 10th Edition [21]. Comorbidity score was calculated according to the Charlson Comorbidity Index based on diagnoses from five years before entering the study [22, 23]. *Hospital activity* included clinical testing by number of MRI-scans, CT-scans EKGs, x-rays and laboratory tests conducted during the patient’s hospital contacts. These were reported as yes/no per contact, for each test completed. *Times to first hospital doctor contact or hospital specialist contact* were measured from time of arrival.

## Statistical analysis

Descriptive statistics were used to summarise characteristics at contact level. Characteristics on continuous variables are shown by median and interquartile range (IQR) due to non-normal distributions. Categorical variables are presented by counts and proportions. Differences in numbers and percent point are displayed between periods (Table 1 and 2).

**Table 1:** Characteristics of all contacts at the hospital from 1st March until 31st of December 2016 (before) and 1st March until 31st of December 2017 (after)

Characteristics at contact level	All^a^ March-Dec 2016/2017	Before March – Dec. 2016	After March – Dec. 2017	Difference^b^ Before-after
All hospital contacts, n	5,339	2,633	2,706	73
Unique patients, n	3,632^a^	1,893	2,033	140
Gender - female, n (%)	2,607 (48.8)	1,294 (49.2)	1,313 (48.5)	(-0.7)
Age, median (IQR)	70 (57:79)	69 (57:78)	70 (57:79)	1
Diagnosis, n (%)^c^				
Circulatory diseases	1,208 (22.6)	536 (20.4)	672 (24.8)	(4.4)
Respiratory diseases	965 (18.1)	506 (19.2)	459 (17.0)	(-2.2)
Infectious diseases	285 (5.3)	163 (6.2)	122 (4.5)	(-1.7)
Digestive diseases	321 (6.0)	168 (6.4)	153 (5.7)	(-0.7)
Endocrine and metabolic diseases	234 (4.4)	118 (4.5)	116 (4.3)	(-0.2)
Bones, muscles and connective tissue diseases	189 (3.5)	86 (3.3)	103 (3.8)	(0.5)
Other	2,137 (40.0)	1,056 (40.1)	1,081 (40.0)	(-0.1)
Comorbidity score, median (IQR)	1 (0:3)	2 (0:4)	1 (0:3)	-1
Triage level, n (%)^c^				
5 – Red	158 (3.0)	63 (2.4)	95 (3.5)	(1.1)
4 – Orange	1,019 (19.1)	486 (18.5)	534 (19.7)	(1.2)
3 – Yellow	1,665 (31.2)	763 (29.0)	902 (33.3)	(4.3)
2 – Green	1,711 (32.1)	914 (34.7)	797 (29.5)	(-5.2)
1 - Blue	10 (0.2)	5 (0.2)	5 (0.2)	(0)
Unknown	776 (14.5)	402 (15.3)	374 (13.9)	(-1.4)
Clinical testing (yes/no pr. contact), n (%)				
MRI-scans	174 (3.3)	112 (4.3)	62 (2.3)	(-2.0)
CT-scans	1,021 (19.1)	520 (19.8)	501 (18.5)	(-1.3)
X-rays	2,404 (45.0)	1,256 (47.7)	1,148 (42.4)	(-5.3)
EKGs	116 (2.2)	71 (2.7)	45 (1.7)	(-1.0)
Blood samples	5,015 (93.9)	2,468 (93.7)	2,547 (94.1)	(0.4)

^a^Some patients entered both the before and after period

^b^Differences in percent points are displayed in brackets, while counts and times are without brackets

^c^Numbers were rounded and do not vertically add up to 100%

**Table 2:** Main outcomes of all contacts at the hospital from 1st March until 31st of December 2016 (before) and 1st March until 31st of December 2017 (after)

Outcomes at contact level	All^a^ March-Dec 2016/2017	Before March – Dec. 2016	After March – Dec. 2017	Difference^b^ Before-after
All hospital contacts, n	5,339	2,633	2,706	73
Hospital length-of-stay, median hours (IQR)^c^	26.3 (5.7: 76.1)	32.6 (8.8: 94.3)	22.3 (4.4: 70.6)	-10.3
0-24 hours, n (%)	2,440 (45,7)	1,015 (38.6)	1,425 (52.7)	(14.1)
24-72 hours, n (%)	854 (16)	471 (17.9)	383 (14.2)	(-3.7)
72 hours-7 days, n (%)	1,619 (30.3)	901 (34.2)	718 (26.5)	(-7.7)
> 7 days, n (%)	426 (8.0)	246 (9.3)	180 (6.7)	(-2.6)
Proportion admitted, n (%)	4,063 (76.1)	2,488 (94.5)	1,575 (58.2)	(-36.3)
Length-of-admission time, median hours (IQR)^c^	47.2 (21.4: 97.3)	44.9 (14.9: 95.9)	50.9 (23.7: 111)	6
0-24 hours, n (%)	1,294 (31.9)	880 (35.4)	414 (26.3)	(-9.1)
24-72 hours, n (%)	786 (19.4)	466 (18.7)	320 (20.3)	(1.6)
72 hours-7 days, n (%)	1,572 (38.9)	897 (36.1)	675 (42.9)	(6,8)
> 7 days, n (%)	411 (10,1)	245 (9.9)	166 (10.5)	(0.6)
30-day unplanned readmissions, n (%)	571 (10.7)	355 (13.5)	216 (8.0)	(-5.5)
30-day re-contacts, n (%)	794 (14.9)	403 (15.3)	391 (14.5)	(-0.8)
Time to first doctor contact, minutes (IQR)	25 (14:39)	27 (15:45)	23 (13:35)	-4
Time to first specialist contact, minutes (IQR)	52 (40:66)	55 (42:71)	50 (38: 62)	-5
30-day contacts to GP, median (IQR)	1 (0:2)	1 (0:3)	1 (0:2)	0
30-day mortality, n (%)	129 (2.4)	72 (2.7)	57 (2.1)	(-0.6)

^a^Some patients entered both in the before and after period (partly repeated measures at patient level within and between periods)

^b^Differences in percent point are displayed in brackets, while counts and times are without brackets

^c^Hospital length-of-stay: the time from arrival to departing either as outpatient or by discharge. Length-of-admission time: the time from admission to the time of discharge

**Fig. 2:**
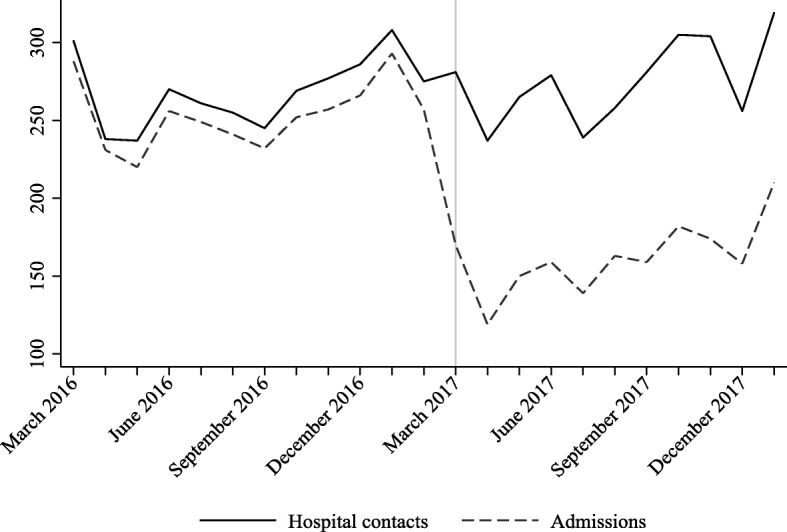
Hospital contacts and number of admissions summed at thirty-day intervals before and after converting inpatient take to outpatient the 1st of March 2017

**Fig. 3:**
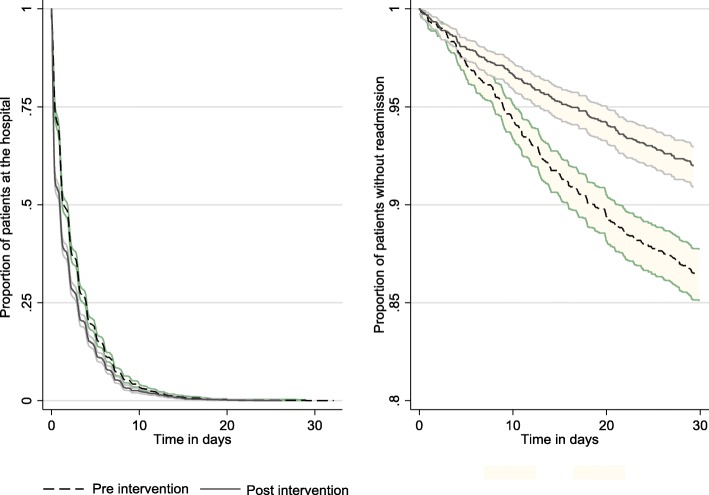
Kaplan-Meier plots of a) proportion of patients staying at the hospital (hospital length-of-stay as outpatient or inpatient) and b) proportion of patients until first unplanned readmission, before and after converting inpatient take to outpatient the 1st of March 2017 with 95% confidence intervals

Hospital contacts and number of admissions were summed at thirty-day intervals and displayed using a time-series graph, Fig. 2. The proportion of patients staying at the hospital (hospital length-of-stay) and the proportion of patients until first unplanned readmission were displayed using Kaplan-Meier plots with 30 days follow-up (Fig. 3).

We dichotomised admission, readmission (yes/no within 4 h to 30 days), re-contact (yes/no within 4 h to 30 days), contact to GP (contact/no contact within 30 days) and mortality (dead/alive within 30 days) and hospital length-of-stay and length-of-admission time at three different levels: 24 h, 72 h, and 7 days. The likelihood of having a long hospital length-of-stay, length-of-admission time, the likelihood of admission, readmission, re-contact, contact to GP and mortality were estimated with a generalised linear model (Table 3), where we used robust variance estimates to account for random effects in patients that contacted the hospital more than once. An adjusted model included gender, age and triage score.

**Table 3:** Unadjusted and adjusted likelihood (prevalence proportion ratio (PPR)) of having a hospital length-of-stay longer than 24, 72 and 7 days, admission, length-of-admission-time longer than 24, 72 and 7 days, re-contact within 30 days, readmission, contact to GP within 30 days, and mortality within 30 days after comparing before and after the intervention (1st March 2017)

	PPR (95%CI)	*p*-value	PPR (95%CI)	*p*-value
	Unadjusted		Adjusted^b^	
Hospital length-of-stay^a^				
> 24 hours	0.77 (0.73: 0.81)	**<0.001**	0.54 (0.52: 0.57)	**<0.001**
> 72 hours	0.76 (0.71: 0.82)	**<0.001**	0.45 (0.42: 0.48)	**<0.001**
> 7 days	0.72 (0.59: 0.86)	**<0.001**	0.70 (0.58: 0.84)	**<0.001**
Admission	0.57 (0.54: 0.59)	**<0.001**	0.57 (0.55: 0.59)	**<0.001**
Length-of-admission time^a^				
> 24 hours	1.14 (1.09: 1.19)	**<0.001**	1.08 (1.03: 1.12)	**<0.001**
> 72 hours	1.16 (1.09: 1.24)	**<0.001**	1.08 (1.02: 1.15)	**0.013**
> 7 days	1.07 (0.89: 1.29)	0.480	0.99 (0.82: 1.20)	0.951
30-day re-contacts	0.94 (0.81: 1.11)	0.480	0.96 (0.82: 1.12)	0.575
30-day unplanned readmissions	0.59 (0.48: 0.72)	**<0.001**	0.59 (0.49: 0.72)	**<0.001**
30-day contacts to GP	0.98 (0.93: 1.02)	0.257	0.97 (0.93: 1.02)	0.201
30-day mortality	0.84 (0.60: 1.17)	0.302	0.81 (0.58: 1.14)	0.232

Robust standard errors were used to adjust for clusters of individuals. Statistically significant values are in bold

^a^Hospital length-of-stay: the time from arrival to departing either as outpatient or by discharge. Length-of-admission time: the time from admission to the time of discharge Dichotomised at 24 hours, 72 hours and 7 days, the likelihood of having a length-of-stay longer than 24 hours, 72 hours, and 7 days.

^b^Adjusted for gender, age and triage score

Data processing was carried out using Stata software version 14 (Stata Statistical Software, College Station, TX).

## Results

Characteristics of all contacts before and after intervention are summarised in Table 1. The number of referrals remained broadly the same with 2633 in the before and 2706 in the after period, amounting to 1893 unique patients prior to (1.39 contact/patient) and 2033 post the intervention (1.33 contact/patient). During the post-intervention period, an average of 1.62 patients was assessed per examination chair per day (during 8 am to 5 pm on week days).

Age, gender and discharge diagnoses were reasonably similar between the two periods although there were an increase in proportion of circulatory diseases and decrease in respiratory diseases. Most patients scored yellow or green in triage in both periods (63.7% before and 62.8% after) (Table 1).

As shown in Table 2, the median hospital length-of-stay (including both outpatients and inpatients) was reduced by 10.3 h (32.6 h before (IQR: 8.8: 94.3) and 22.3 h after (IQR 4.4: 70.6)). The group of patients who had a hospital length-of-stay between 0 and 24 h rose by 14.1% after the index date for the intervention while lowering the number of patients categorised with longer stays. The proportion of contacts leading to admissions dropped by 36.3%-points (94.5% before to 58.2% after). For patients admitted, the median length-of-admission time rose by 6 h (44.9 h before to 50.9 h after). A higher proportion of admitted patients had a length-of-admission time > 24 h after converting to fast track outpatient assessments. The proportion with 30-day unplanned readmissions was reduced by 5.5%-points (from 13.5% before to 8.0% after), while 30-day re-contacts stayed the same across periods. The time to first doctor contact, regardless of specialist level, was almost the same before and after. Median number of contacts to GP and number of deaths stayed the same across the periods.

In Table 3 *showing prevalence proportion ratios (PPR)*, the likelihood of a total hospital length-of-stay (including both outpatients and inpatients) longer than 24 h, 72 h and seven days was statistically significantly lower after the intervention compared to before. For patients admitted, the risk of having a length-of-admission time for more than 24 (compared to less than 24 h) and 72 h (compared to less than 72 h) increased statistically significantly after the change, but not for stays over 7 days (compared to less than 7 days). The likelihood of unplanned readmissions reduced after the change (adjusted PPR = 0.59, 95%CI: 0.49: 0.72). Re-contacts, death after 30 days, and having contact to a GP during 30 days were similar before and after the intervention.

Figure 2 illustrates the development in monthly contacts and use of admissions. Note the strong decrease in the number of admissions at the time of the intervention being implemented. In Fig. 3, we show that the vast majority of patients had a hospital length-of-stay of less than a weeks time and that patients who were readmitted stayed rather proportional in pattern over 30 days time from leaving the hospital.

The average monthly medical bed occupancy for all admitted patients (including transfers, elective and scheduled) at the wards ranged from 80 to 96% prior to the index date (March-Dec.) and from 88 to 105% in the following ten months from the index date with 8 fewer beds substituted with 8 outpatient fast-track examination chairs.

## Discussion

In this before-and-after study of 5339 acute hospital contacts, we found that admissions markedly decreased after shifting to an outpatient take with fast-track assessment of acute medical patients. Additionally, the median hospital length-of-stay was reduced with almost half a day. The median admission time rose as expected after commencing the acute outpatient take, as the remaining admitted patients in the after-period are more likely to have had a greater medical need and morbidity. We saw that readmissions decreased while the likelihood of re-contacting the hospital, contacting GP and mortality stayed the same across periods. Bed occupancy ranged higher with fewer beds during the post-period for all admitted patients, although, this could be contributed by the elective, scheduled, and transferred patients who accounted for almost half of the contacts.

To our knowledge, no former studies resemble the present intervention, converting entire wards to an outpatient acute medical take while undergoing fast track assessment in a hospital examination chair. The constant flow of patients using the special medical examination chairs and beds, challenges the current way of looking at bed occupancy and capacity, as several patients occupy the beds/chairs during the day. However, several studies have converted inpatient trajectories to outpatient [14] and several studies have tried to solve the burgeoning problem of acute take and overcrowded hospitals e.g. by leading acute patients through assessment units instead of direct admission in a medical or an emergency department [9, 10, 14, 24, 25]. Instead of introducing new functions and extra resources, our intervention used the existing resources and competences but changes the framework for using these.

Moeller et al. described how a 24-h outpatient take was implemented at the Silkeborg Regional Hospital allowing patients to call at any time when experiencing exacerbation of symptoms related to their chronic disease. This significantly decreased the use of admissions by approx. 40% for some diagnoses [15]. The intervention and participants described in Moeller et al. appears in our current study and constitutes approx. 10% of our study population. These patients lower the number of admissions in both before and after intervention periods as they are received as outpatients in the wards and add to a low hospital length-of-stay. These patients are allowed open hospital access and constitute a selected group with a chronic disease, whereas we include all acute medical patients referred.

Existing studies show positive results with earlier senior clinician involvement, which has shown to reduce the length of hospitalisation [26]. In the current study, the same professionals examine the patients on arrival before and after the intervention while a one-hour deadline was introduced after the intervention. Within this deadline, the nurses and the younger physicians must have completed triaging and examinations, and assessment of test results. The specialist-physician must also have decided whether the patient should be admitted and have made a plan for investigation and treatment.

Hospital readmission is reported as a post-discharge adverse outcome from admission and knowledge is limited on how to change the pathway for acute medical patients in order to overcome readmissions [9, 13]. Many factors have been highlighted as contributors to increased rates of patient readmission, and a common cause includes accelerated turnovers, which does not relieve the problem of overcrowded hospitals [27, 28]. A systematic review of 34 studies on readmissions deemed avoidable, found the median proportion of preventable readmissions was 27% ranging from 5 to 79% [27]. According to our study and the study by Moeller et al., admissions may be prevented by converting to an outpatient take, our study also showed that readmissions are preventable.

Conley et al. found in outpatient management strategies, that several acute medical conditions had no significant difference in mortality, compared with inpatient admission [14] .Reviews with an outpatient strategy were targeted single medical conditions, which contrasts with this study. For quick diagnostic units, the evidence found by Conley et al. demonstrated lower mortality rates. For observation units, several acute medical conditions were found to have no difference in mortality and a decreased length-of-stay compared to inpatient admission [14]. Although our intervention did not include an assessment unit before entering a medical ward, our results are in line with previous studies, suggesting no difference in mortality.

### Strengths and limitations

This study included all acute patients referred to medical wards and had statistically precision due to a large number of hospital contacts. Due to the extensive electronic registration, our data can be considered of highly valid and complete. With caution, our findings can be generalised to other health care systems. Several limitations merit consideration. These are results based on a short observation period. The before-after study design runs the risk of conclusions being made that actually result from temporal trends and challenges in regard to sustainability and generalizability. We accounted for a random patient effect in the analysis in Table 3 due to non-independency intra and inter periods. It seems unlikely that seasonal variation in climate may have produced the results, as the change was seen from the date of intervention. Missing data from winter months following the intervention limits the generalisability. Presumably, an increased number of patients during winter months could lead to a delay in assessments and as a result, increased hospital length-of-stay. Also, it is difficult to draw any conclusion with regard to readmissions and contacts to GPs because being admitted leaves less time to be readmitted and to seek the GP. However, as we saw a decrease in total admission time, the findings seem robust. Still, our intervention allows for a closer follow-up as outpatient, which may result in substituting GP contacts.

### Implications

The innovative way of receiving acute patients in this study avoids adding processes to the patient pathways as patients are assessed directly in the internal medical wards. This bridges the out- and inpatient occupancy in contrast to traditional systems that already utilize emergency rooms or observation units. However, one may argue that our examination areas in many ways involve the same principle as traditional models, assessing patients in arranged areas to the needed level of care and evaluating whether admission is required. Our hospital has a small acute take and is a development hospital with an exceptional collaboration between staff within and between wards. The very well-functioning infrastructure may contrast to other models. However, the main contrast to the traditional take through an emergency department or an observation unit is the fact that this hospital only receives acute patients who are referred and with medical issues. This model used direct access to a mandatory specialist staffed fast-track outpatient internal medicine assessment, avoiding unnecessary time consumption compared to previous models. Moreover, the outpatient take allows for a dynamic occupancy as the medical examination chairs are constantly occupied by different patients during the day.

To assume the same benefits will apply in other hospitals should be made with caution. We argue that elements of the model of receiving acute patients as outpatients with fast track assessment might be incorporated in different models. The results of this study indicate that the model has the potential to reduce the consistently increasing pressure on hospital services. However, more studies are needed to asses if an outpatient take with fast track assessment is sustainable in different settings. A randomised trial including the same patient groups and clinicians with the same level of specialisation and experience would be an ideal design for assessing this. Furthermore, studies should also cover the patients and the healthcare professionals’ experiences along with the cost-effectiveness of an outpatient acute take in medical wards.

## Conclusion

This study provides evidence that after introducing an outpatient take instead of admitting all referred acute patients as an integrated part of the triage, the number of admissions and readmissions and the hospital length-of-stay decreased, leaving the admission time, as expected, slightly increased for the remaining patients who were admitted. This study indicates that a relatively simple organizational change in the form of placing acutely referred medical patients in an examination chair as an outpatient and carrying out a fast track examination and treatment program can assist in meeting the challenge of overcrowded hospitals for “ordinary” acute referred patients.

## Abbreviations

CT: computed tomography
EKG: electrocardiogram
GP: general practitioner
IQR: interquartile range
MRI: magnetic resonance imaging
PAS: patient administration system
PPR: prevalence proportion ratio
PRN: personal registration number
